# Supplementary material for: Competency analysis based on accounting career anchors using clustering techniques
Source: PLoS One. 2023 Jan 6;18(1):e0279989. doi: 10.1371/journal.pone.0279989 (PMC9821735; doi:10.1371/journal.pone.0279989)
Supplement: Attachment — Submitted filename: Response_Letter.pdf [file pone.0279989.s001.pdf]

Subject: Submission of revised paper (PONE-D-22-01029)

June 18, 2022

Sina Safayi, D.V.M., Ph.D.
Plos One

Dear Editor

Thank you for allowing us to submit a revised draft of our manuscript titled "Competency analysis based on accounting career anchors using clustering techniques." We appreciate the time and effort that you and the reviewers have dedicated to providing your valuable feedback on our manuscript.

We are grateful to the reviewers for their insightful comments on our paper. We have been able to incorporate changes to reflect most of the suggestions provided by the reviewers. We have highlighted the changes within the manuscript. We are sure that our paper's quality and legibility have significantly improved, thanks to the reviewers' comments.

Here is a point-by-point response to the reviewers' comments and concerns.

Reviewer 1

1.1 —

The authors discuss the disconnect between what students may be taught at educational institutions vs the realities of actual on-the-job performance, worker expectations, and career anchors. The authors specifically look at the profiles of competencies characterizing the professional accountant, using data taken from Peruvian and Colombian participants.

The authors have given a good overall review of career anchor theoretical frameworks and recent literature addressing these anchors. The authors describe their method of analysis and clustering particularly well. I also enjoyed the description of the ideas articulated in the introduction and the overall concluding thoughts. However, I think the paper is not fully understandable in its current form. But it has potential to be very interesting once the authors have undertaken revision of the article, and I encourage the authors to bear in mind how interesting this work could be to non-specialists, particularly in taking greater care to explicitly point out the steps taken, and the meanings of the numbers in Table 6. This then has the potential to be much more accessible to those who work in career development spaces but do not have expertise in clustering.

Reply: Dear reviewer, we appreciate your positive comments about our manuscript. The improvements are reported below:

1. In the first section, Schein and Brooks' theory concerning running anchors was added to the introduction, answering the following questions: What are running anchors? What is the importance of running anchors?

2. A more detailed description of the data in Table 8, which summarizes the results of our study, was added to the results section.

1.2 — What exactly do the authors mean by an "academic cycle"? From Table 4, it is equated with "Level", and refers to the students academic period - is this a standard measure of time, such as a semester? Or does this time period vary between campuses e.g. is it possible it could refer to a trimester in one and a semester in another? Making this clearer would be helpful..

Reply: It is the academic period of 6 months of university studies where the student develops the teaching-learning process.

1.3 — The sample sizes for Peruvian vs Colombian participants are not explained, what is the reason for the discrepancy in sample size and do the authors think that anything is affected in their overall analysis by this?

Reply: The total number of accounting students in both countries was considered in both samples; this means that the number of students in Peru was higher because it had three university campuses and only one in Colombia. This is detailed in Tables 3 and 4.

1.4 — How do the authors define which are the anchors that are most important based on the numerical values in Table 6? Line 211 states "whose outstanding professional anchors are entrepreneurial creativity, service, and pure challenge." In the table, these are the 3 highest numerical values, but the number are 23, 22, 22 respectively. Autonomy, Stability and Lifestyle all have values of 21 - why are these not also important? Especially as for the 51-yer old male, values of 21 are cited (for Services) as important. The authors could explain more clearly how the numbers in Table 6 are converted into the conclusions they provide.

Reply: Considering your observation, the predominant career anchors in the manuscript's text were expanded, with a minimum value of 21 for each anchor. Therefore, the following anchors were added to the Peruvian sample: functional technician, autonomy, stability, and lifestyle. In the case of Colombia: functional technician, autonomy, and pure challenge.

1.5 — Given that PLOS ONE does not copyedit articles, some work needs to be undertaken to clarify the English, particularly the grammar which is understandable in the main, but not standard. For example, line 35, "do not limit the research. Such as..." should probably read as "do not limit the research, such as". Another example is on line 39, "Include a diversity of groups, with multiple characteristics 39 [8]" does not make grammatical sense; likewise on line 73 "students who were not enrolled in the academic semester conducted the survey" presumably means "students who were not enrolled in the academic semester when the survey was conducted." There are also other proofing errors such as "The of this research was..." on line 28, for example. Further examples: The sentence on line 218 beginning "Pure challenge anchor" does not make grammatical sense; likewise in line 224 "it can be identified a male model" does not make grammatical sense. Line 274 reads, "The explanation of this anchor is found in Section ." The name of the section being referred to is missing. The first sentence in the Conclusion does not make grammatical sense. Overall the paper was mostly understandable and I was generally able to follow the logic and arguments,

but not always, and I think this affects the impact the paper could have. One exception was the results section, where I was initially unable to follow the discussion in "Peruvian university campus: Description of cluster significance". For example, on line 212, a sentence begins "Both clusters. . . ", but only one cluster has been discussed clearly so far, and so the reader cannot easily determine which two clusters are being referred to. It was only when I was reading the Colombian results that I understood that both the Peruvian clusters are what is being compared - the section does begin by saying this but it was not initially clear. It is possible that this easily could be much clearer by moving the paragraph beginning on line 224 to come before the sentence beginning "Both clusters" on line 212.

Reply: Thank you very much for your detailed comments. We have made all the suggested changes, and the manuscript has been sent to AJE for proofreading.

Reviewer 2

2.1 — PONE-D-22-01029 titled "Competency analysis based on accounting career anchors using clustering techniques" by Avila-George et al., provides a quantitative method to analyze and compare career anchors (competencies) influencing aspirations and identity formation, by using test cases of future accountants in Peru and Colombia. The study highlights differences in values between professionals of the two countries with similar educational background, indicating influence of socio-economic cultures in career choices. The inherent merit of this study is its inclusion of diverse international perspectives and diversifying representative data in analyzing career anchors. However, the authors need to do a better job highlighting the broad significance of this study. I recommend approval with following suggestions and considerations for revisions and improvement Major comments

Highlight the significance and purpose of study better. It was hard to comprehend the purpose, focus and broad impact of this study until reading much of the introduction. There is no mention of the purpose or significance of the study in the abstract either. The paper is focused on methodologies far more than outlining the problem statement and the merits of investigating career anchors internationally. It's primarily written for niche readers well versed with the field and literature (Schein and Brooks) and needs to expand its communication to include non-specialists.

Reply: The abstract was improved according to your suggestions; it considers the impact of the study and the significance of the proposal.

2.2 — After reading the paper, I am curious whether this methodology can be used to cluster career anchors for longitudinal career progression analysis by country. For example- does career anchors and career orientations change for students (future accountants) after they spend a few years in the workforce due to influence of economic and marketplace environments?

Reply: The research is cross-sectional, but it can be applied to other samples and not necessarily to professional accountants since this survey is for all types of professionals. The predominant anchors obtained in this study should not necessarily be the same in another period since the results of this study cannot be generalized to other case studies.

2.3 — The authors mention skills gap and inadequate higher education training to meet the skills gaps. Can this methodology be used to define said skills gap in accounting profession by country. For example- in parallel to career orientation and anchors of students, understanding profile of career anchors and competencies by analyzing job descriptions and labor data for accountant positions in those countries will highlight clusters of shared values and disparities.

Reply: No, this study only develops predominant anchors in a professional profile. What you can suddenly make some comparison would be of these anchors with the competencies demanded in the labor market.

2.4 — The manuscript requires proofreading. There are regular typos and missing words.

Reply: The manuscript has been sent to AJE for proofreading.

Reviewer 3

3.1 — There were numerous grammatical and vocabulary usage errors, which in some cases requires the reader to guess at the point being made.

Reply: The manuscript has been sent to AJE for proofreading.

3.2 — There were no p values reported with the ANOVA analysis.

Reply: The p-value values were added to Table 9.

3.3 — The survey questions are not available.

Reply: It can be found at the following link: https://github.com/jasg1612/anchors

3.4 — The survey questions are not available.

Reply: In both samples, the total number of accounting students from both countries was considered.

Table 1: **Sample in Peru**

**Headquarter**	**Female**	**Male**	**Total**
Juliaca	206	112	318
Lima	50	37	87
Tarapoto	39	15	54
Total	295	164	459

Table 2: **Sample in Colombia**

**Headquarter**	**Female**	**Male**	**Total**
Principal	34	30	64

We hope the revised version fulfills the expectations of the editorial team and look forward to hearing from you in due course.

Sincerely,

Dr. Himer Avila-George
Professor
Departament of Computer Science and Engineering
University of Guadalajara, Mexico
E-mail: himer.avila@academicos.udg.mx

On behalf of all authors.
