# Supplementary material for: Competency analysis based on accounting career anchors using clustering techniques
Source: PLoS One. 2023 Jan 6;18(1):e0279989. doi: 10.1371/journal.pone.0279989 (PMC9821735; doi:10.1371/journal.pone.0279989)
Supplement: Attachment — Submitted filename: Response letter.pdf [file pone.0279989.s002.pdf]

Subject: Submission of revised paper (PONE-D-22-01029)

November 16, 2022

Sina Safayi, D.V.M., Ph.D.
Plos One

Dear Editor

Thank you for allowing us to submit a revised draft of our manuscript titled "Competency analysis based on accounting career anchors using clustering techniques." We appreciate the time and effort that you and the reviewers have dedicated to providing your valuable feedback on our manuscript.

We are grateful to the reviewers for their insightful comments on our paper. We have been able to incorporate changes to reflect most of the suggestions provided by the reviewers. We have highlighted the changes within the manuscript. We are sure that our paper's quality and legibility have significantly improved, thanks to the reviewers' comments.

Reviewer 1

1.1 —

The clusters in Table 8 show that for Peru, both clusters are represented by the same gender (I am guessing male, but it isn't clear because the scoring isn't defined). Can the authors describe what this means? What "happens" to females in the sample?

Reply: Derived from their punctual comments, the experimentation has been redefined with the search for the best hyperparameters used by the algorithms to cluster the data. The algorithms were compared using the Silhouette coefficient metric, which measures the level of cohesion of the clustered data. Thus defining the best clustering algorithm. The result of this comparison was that the DBSCAN algorithm obtained the best measure of cohesion, as described in Table 8, in both samples from Peru and Colombia. Once the DBSCAN algorithm labeled the datasets' rows, each cluster's mean was calculated. Table 9 shows the summary of the labeling of the data from cluster 0 of each of the two datasets; the data from cluster -1 for each dataset were discarded because the number of records was not representative for calculating a mean (i.e., these data could be considered as noise), see Figures 4 and 5.

Clustering in the present work was used for the dimensional reduction of the data, in this case, applied to reduce the number of career anchors to the most prevalent anchors in each sample of Peru and Colombia. The latter process was validated by the Fisher's F statistic of ANOVA, which measures the correlations between the career anchors with cluster 0, identifying by the estimated value those with the highest value that was prevalent in the clusters.

On the other hand, the female gender does not appear in any of the clusters because the male gender was prevalent among the respondents.

1.2 — Another interesting aspect that is apparent from Figures 4 and 5 and Table 8 is that one cluster has scores that are all lower for the anchors than the other cluster. Is this expected? Would it not be possible that clusters would emerge with each having anchors higher than the other cluster? It would be helpful to see this approached in the discussion.

Reply: The scores of the anchors will depend on the values of the group of records labeled by the cluster, since the anchors were averaged and this measure is susceptible to the values of the anchors at the time of averaging. The DBSCAN algorithm detected cluster 0 and -1. Cluster -1 had a minimum number of records belonging to the cluster, therefore this cluster is discarded, leaving only the only cluster 0, observing the prevalences in this cluster 0. The idea of the research was the dimensional reduction of the career anchors therefore a representative cluster was expected in the whole sample defining the prevalent anchors in each sample, by the mean scores defined in each anchor.

1.3 — It is still not clear what is meant by an "academic cycle" in the text of the paper - this needs to be explained thoroughly. In response to my previous review, the authors replied to me that, "It is the academic period of 6 months of university studies where the student develops the teaching-learning process." I'm still not clear what this means; how many 6-month periods are there in a student's course? Are there 2 periods in a year, or does each cycle represent a new year? Perhaps there needs to be a short explanation of the structure of the accountancy courses in the introduction to make this clearer. Another alternative that the authors could consider - are the cycles relevant to their conclusions? The cycles are only mentioned as an additional characteristic but I don't think there is any discussion of the significance. Are later (e.g. 4/5) more important? Is there a distribution of recipients across cycles? If it's not important to the author's conclusions, perhaps all mention of cycles could just be removed. Either way, I am still confused by the concept and so am concerned many readers will be too.

Reply: We understand your doubts, you are right that the way the article is written causes confusion. Therefore, it was considered pertinent to eliminate the term academic cycle, since the anchors are part of the academic formation of the students throughout their stay at the university.

1.4 — One minor edit needed is that the Figures are not comprehensively referenced in the text; for example, it is not trivial to find the text that aligns with Figure 4 in order to appreciate the context of the Figure. Figure 5 is mentioned however.

Reply: The figures were improved according to the explanation in Table 9; the boxplots describe the data interval of each cluster and the mean described in the table. The reference to figure 4 was added on line 234, its explanation

1.5 — Another minor edit related to the figures is to add text to the figure legends to clarify definitions/colors in used. One example: which colors in Figures 4 and 5 refer to the anchor? What do the diamonds denote? Please include explanations in the figure legends. Another example: in the figure legend for Table 8, it should be explained clearly what '1' means for gender, and what '4' means for academic level. While it's possible to figure this out from the main text, the figure legend should contain all of the information so that anyone can look at the figure, read the legend, and understand all of the information contained within it, without having to hunt through the text to find an explanation. I think the significance of the author's work will be much more readily

apparent with greater clarity about what the figures describe in the text of the figure legends. Lines 275 to 286 are an example of where this work happens in the main text; but the figure legend should also allow the reader to come to this conclusion themselves, so that they are able to agree with the authors' conclusion in lines 275 to 286.

Reply: In response to their recommendations, the legend to Table 09 has been improved, explaining the numbers appearing in the age column, and a legend has been added to Figures 4 and 5, explaining the boxplot colors, the points outside the interval.

1.6 — Once more, with relation to the figures - the y axes should be defined to describe what the numbers signify. And the y axes in Figures 4 and 5 differ within the figure for each cluster; they should be the same in order to allow the comparison that the figure begs.

Reply: The Y-axis values in Figures 4 and 5 have been standardized; a legend has been added to both Figures explaining the meaning of the Y-axis values.

1.7 — Lines 212 and 302 - The name of the Section in which the explanation can be found is missing.

Reply: The cross-reference has been corrected in both sections.

Reviewer 2

2.1 — The aim of the study as defined in the abstract and introduction is not consistent with the chosen survey instrument. The abstract states that "This study aims to define the career anchors of accounting students and the skills and knowledge required to be learned during professional training." The introduction explains the aim like this: "The aim of this research was to analyze the profile of competencies that characterize professional accountants." Although the career anchors are discussed in detail, there is no mention in the paper of skills development, training program requirements, or competencies.

Reply: The wording of the objective presented in the introduction has been improved to be consistent with the study conducted and all sections of the paper.

This research work aims to identify the prevalent anchors in the professional accounting career using the Schein scale and to describe the prevalent anchors by defining the values, attitudes, aptitudes, skills, and interests and represent core elements in forming professionals.

2.2 — These questions are integral to understanding a trainee's workplace preferences and long term career goals, and the survey is a important tool used by career counselors, but, it is my opinion that this survey, in and of itself, does not represent a novel data collection tool that could form the basis of a peer reviewed paper. Nor do the questions allow any conclusions about training competencies or accounting-specific knowledge.

Reply: The instrument is based on José Medina's adaptation of Schein's anchor theory, directing each question to each concept and purpose of the anchor. The author of the book: Lead your career: Don't

let others decide for you has extensive experience in personnel selection and research in companies, has a doctorate in psychology, and is an expert in Organization Development from the National Training Laboratories Institute in Washington D.C. This shows us that the instrument has a theoretical foundation on the side of Schein's anchors, it is based on the judgment of the expert José Medina. Crombach's alpha statistical reliability test was also performed with a result of 0.94 for the instrument. In this sense, the study's purpose is to understand students' workplace preferences and long-term career goals.

2.3 — To advance the field of accountant training and career development it is important to present data that will inform suggestions about program graduates' career readiness, knowledge acquisition, employment outcomes, performance, etc. If the purpose of the paper is to show how clustering algorithms can help researchers to better understand survey responses, then this should be stated as the aim of the study. And if this is to be the case, the rationale for the methods used needs to be explained more thoroughly. For example, using three clustering methods (KMEANS, DBSCAN, and BIRCH) without an a priori rational and then stating simply that DBSCAN was chosen because it "performs best" (line 216) with no further explanation leads this reader to conclude that the authors have not made themselves experts in clustering methods.

Reply: The purposes of the study were explained in the introduction, the abstract and in the exploratory analysis section. To achieve this purpose, the explanatory research was used, which is based on the dimensional reduction of the variables that explain the case study, which consists of the career anchors of professionals in accounting, by reducing the dimensions, the prevalent anchors in the student's training were found, being able to make the corresponding analysis then of the skills found, aptitudes, attitudes developed in such training. After performing the dimensional reduction of the anchors with the clustering, a second statistical test was performed to cross the information of the clusters with the ANOVA test and to explain the prevalent anchors.

2.4 — Another aspect of the paper that is not clear is why there are only two clusters assigned? How were those clusters defined? Can average scores for each career anchor be reported for each cluster? Perhaps this is what table 8 is attempting to describe, but the columns for the career anchors are described in the methods section as being the sum of all group records (line 222), but the numbers are all less than 30 when hundreds of surveys were recorded. Similarly, reporting averages for each career anchor in each cluster would be more valuable than choosing one representative individual in each cluster and reporting about their survey responses as is done in the lines 266-274.

Reply: Another aspect of the document that is not clear is why only two clusters are assigned? How were these clusters defined? Hyperparameter tuning, described in Table 07, was performed using the cohesion metric, silhouette, with the best algorithm determining the number of clusters using the optimized hyperparameters Can the mean scores of each run anchor be reported for each of each cluster? The values that were averaged were those assigned in each anchor, product of the sum of the questions related to each anchor then were these values averaged taking into consideration the cluster that was labeled with anchor 0 and anchor -1, the records were eliminated for representing a small amount. This average value was below the value of 30, since most of the values were below 30.

2.5 — Table 9 is also not well-described in the methods section or the results section. The authors use the data in Table 9 to draw a conclusion about the imagination and innovative abilities of the

survey respondents (line 257), but as stated above, the survey does not address competencies, skills, or proficiency from a self-reported lens, and definitely not from an impartial external lens.

Reply: Clustering was used to reduce the dimensions of the data; in this case, it was applied to find the most prevalent anchors in each sample from Peru and Colombia (the purpose of the study). This process was validated by the Fisher's F statistic of ANOVA, which measures the correlations between the anchors run with cluster 0, identifying by Fisher's estimated value those with the highest prevalence value in the cluster. In the methodology, the exploratory analysis and statistical test section explain how ANOVA works with clustering as a statistical tool to confirm the dimensional reduction analysis of clustering.

2.6 — Other elements of the paper that would need to be fixed include multiple instances where sections are referred to without section numbers (e.g. Line 212, 268, 302). The survey response rate for both locations should be reported. Table 5 contains multiples of question numbers in the same anchor categories.

Reply: Added cross references to lines 212, 268, 302 and corrected items in table 05

We hope the revised version fulfills the expectations of the editorial team and look forward to hearing from you in due course.

Sincerely,

Dr. Himer Avila-George
Professor
Departament of Computer Science and Engineering
University of Guadalajara, Mexico
E-mail: himer.avila@academicos.udg.mx

On behalf of all authors.
